# Scope of rehabilitation for patients with long COVID symptoms in Bangladesh

**DOI:** 10.3389/fresc.2025.1572351

**Published:** 2025-05-15

**Authors:** MD. Waliul Islam, Ehsanur Rahman, K. M. Amran Hossain, Md. Zahid Hossain

**Affiliations:** ^1^ Centre for the Rehabilitation of the Paralysed, Dhaka, Bangladesh; ^2^ Jashore University of Science and Technology, Jashore, Bangladesh

**Keywords:** C19-YRS, long COVID, phenotypes, post-COVID-19 condition, SARS-CoV-2, rehabilitation and physiotherapy

## Abstract

**Background:**

The Bangladeshi healthcare system had planned to meet the long-term rehabilitation needs of people who had suffered due to COVID, as well as those whose health and level of activity had declined during the COVID pandemic. The goal is to apply the COVID-19 Yorkshire Rehabilitation Scale (C-YRS) to ascertain the number of health domains in which a person with PCS should undergo rehabilitation.

**Methods:**

We carried out a quantitative cross-sectional study. The eight administrative divisions provided the pool of participants for selecting the 409 people comprised by the stratified sampling. We collected data using a semi-structured questionnaire that included sociodemographics, a symptoms checklist, and the C-YRS.

**Results:**

The most common post-COVID symptoms among the participants were fatigue (34.3%), muscle pain (20%), and dyspnea (12.2%). The comparison between pre- and postinfection symptoms revealed a notable increase in symptom severity and functional impairments. The study also found a significant relationship between symptom severity and place of residence (*p* < 0.5). The study found that the severity of symptoms was mild (30.5% (*n* = 90), moderate 55.3% (*n* = 186), and severe 14.2% (*n* = 94). We also looked for correlations between symptom severity, functional impairment, and health. It showed a significant positive correlation between symptom scores and functional difficulty scores (0.889, *p* < 0.001), while there was a significant negative correlation between symptom scores and overall health (−0.658, *p* < 0.001).

**Conclusion:**

Severity phenotypes can aid in the stratification of people with PCS for targeted therapies and rehabilitation care planning.

## Background

The COVID-19 pandemic has emerged as a substantial public health concern worldwide. The SARS-CoV-2 virus, which originated in Wuhan, China in December 2019, is responsible for the emergence of COVID-19. As of 23 December 2023, the global tally of confirmed COVID-positive patients reached 772,838,745, with a total of 6,988,666 deaths reported (1). According to the Directorate General of Health Services (DGHS), Bangladesh has recorded a total of 2,046,049 confirmed cases of COVID-19, with 29,477 fatalities (2). Additionally, 83.49% of the country's population has received a two-dose vaccine. In Bangladesh, 98% of individuals who have contracted COVID-19 have successfully recuperated (3). However, 16.1% of these individuals continue to experience long-term COVID symptoms even after 31 weeks of recovery. According to a previous study, COVID-19 survivors commonly experienced fatigue, pain, dyspnea, and persistent cough as the most prevalent symptoms of long COVID (4). In a cohort study, individuals afflicted with long COVID experienced enduring and widespread symptoms affecting multiple systems involvement (5). Despite a recuperation period of 7 months, a significant number of individuals stated that they had not regained their previous level of working capacity (5). Individuals with many preexisting comorbidities experience substantial impacts on their quality of life as a result of persistent COVID symptoms (6). This research was imperative to determine the viability of long-term rehabilitation assistance for individuals experiencing signs of long COVID. This was especially apparent in low- to middle-income countries such as Bangladesh, where the majority of the population lives in rural areas outside of Dhaka, the densely populated capital city. Countries are devising strategies to efficiently meet the ongoing rehabilitation needs of those who have suffered negative consequences from COVID-19, as well as those whose health and physical capacities have declined due to the pandemic. Ensuring the availability of services is vital to prevent the negative impact of the critical pandemic response on the outcome (7). We created the Yorkshire Rehabilitation Scale to assess the current level of symptoms in patients with long COVID. This scale facilitates the link between the rehabilitation requirements of COVID-19 patients and the intensity of their symptoms. This applied to both individuals who had already received a diagnosis and those who had not yet received one (8). It was imperative to assess the significance of rehabilitation services for individuals displaying COVID-19 symptoms within the Bangladeshi community, regardless of their COVID status or country. This research study aimed to examine the necessity for rehabilitation among those with long COVID symptoms, particularly those actively seeking rehabilitation.

## Methodology

The study employed a cross-sectional design and followed a quantitative research approach. We collected data from all divisional centers and the CRP headquarters, where individuals with long COVID symptoms are receiving neuromuscular rehabilitation. The study period spanned from 1 August 2021 to March 2022. We calculated the sample size using scientific sampling estimation and chose the standard sample number as a guide for calculation. The Centers for Disease Control and Prevention also verified the sample size using Epi Info software.

The final study sample consisted of 409 individuals, with a prevalence of *P* based on a comprehensive literature review (9), and a population of 166,231,089 in Bangladesh.

### Study procedure

Sampling refers to the process of determining the number and characteristics of participants who participated in a study. This study employed a stratified random sampling technique, which is a sampling method that divides a population into smaller subgroups known as strata. Stratified random sampling, also known as stratification, produces strata based on shared features or characteristics among individuals, such as geographical area (10). The current study selected a rehabilitation center from each division as a stratum. Within each stratum, participants who met the inclusion criteria were selected for interviews. Sampling was conducted within a specific time frame and continued until the targeted sample size was achieved.

### Questionnaire

Using the C19-YRS, researchers collected demographic data, medical history, and information on 16 key symptoms of PCS [including breathlessness, persistent cough, fatigue, pain or discomfort, cognitive problems, anxiety, depression, symptoms of posttraumatic stress disorder (PTSD), palpitations, dizziness, weakness, and sleep problems] as well as their impact on five daily functions (communication, mobility, personal care, wider activities of daily living, and social functions) (11). Respondents rated each symptom or functional ability on a scale of 0–10 (0 being no presence of symptom and 10 being most severe and life disturbing). A 0–10 numerical rating scale (NRS) was also used to assess overall health. Respondents were also asked to rate their pre-illness, which was unique to the C19-YRS and this study. In a group of hospitalized Italian patients, the C19-YRS was a useful patient-reported outcome tool for screening, measuring severity, and monitoring the persistence of symptoms after 12 and 26 weeks post-SARSCoV2 infection. When compared with other existing scales, the selected single items of the scale revealed strong construct validity, indicating its effectiveness in capturing the multisystem state. The measure also assisted in identifying multifaceted rehabilitation needs (physical and psychological). A full biopsychosocial examination can inform individualized intervention. A bigger sample size requires more study on construct validity and responsiveness. A sample was taken from both hospitalized and non-hospitalized patients. Furthermore, the Italian version of the C19-YRS requires a cross-cultural translation.

## Ethical consideration

The proposal was submitted to the Institutional Review Board (IRB) of the Bangladesh Health Profession Institute (BHPI), and after the defense, the research proposal approval was taken from the IRB (CRP/BHPI/IRB/11/2021 I 512). Written consent was taken from each participant before collecting the data. The World Health Organization (WHO) guidelines were always followed to conduct the study. This study was also registered under the WHO trial registry (CTRI/2021/11/038158-India). Informed consent was used to obtain permission from all participants. Participants' rights and privileges were ensured. All the participants were aware of the aim and objectives of the study. The findings of the study were disseminated with the approval of regarding authority.

## Result

### Sociodemographic and clinical information

In this study, out of 409 participants, 59% were male and 41% were female. Only 62 out of 409 were admitted to hospital or received consultation due to long-term COVID symptoms since the pandemic declaration. Approximately half of participants (175) responded that there was a history of COVID in their family. The mean age of the participants was 37.62 years (SD 13.19). Moreover, 96% of participants were vaccinated, but only 24% completed their third dose of vaccination. All sociodemographic information is presented in Table 1.

**Table 1:** Long COVID characteristics according to demography.

Variables	Subcategory	ALL		Mild		Moderate		Severe		*P*
		(*N* = 409)		(*N* = 154)		(*N* = 158)		(*N* = 97)		
Age^a^	Mean ± SD	37.62	±13.19	36.7	±12.99	37.59	±13.1	39.1	±13.69	0.675
Gender^b^	Male	242	59%	98	64%	91	58%	53	55%	<0.001***
	Female	167	41%	56	36%	67	42%	44	45%	
Hospitalization^b^	Hospitalized	62	15%	11	7%	23	15%	28	29%	<0.001***
COVID in community^b^	Yes	248	61%	86	56%	91	58%	71	73%	<0.001***
COVID in family^b^	Yes	175	43%	62	40%	70	44%	43	44%	<0.001***
Vaccination^b^	Non-Vaccinate	16	4%	6	4%	8	5%	2	2%	<0.001***
	1st dose	44	11%	15	10%	17	11%	12	12%	
	2nd dose	251	61%	93	60%	101	64%	57	59%	
	3rd dose	98	24%	40	26%	32	20%	26	27%	
Place of residence^b^	Urban	41	10%	20	13%	17	11%	4	41%	<0.001***
	Rural	160	39%	73	47%	57	36%	30	31%	
	Semi urban	208	51%	61	40%	84	53%	63	65%	

^a^ One-way ANOVA.

^b^ Friedman's ANOVA among mild, moderate, and severe cases with significant values (*P*) as <0.001***.

**Figure 1:**
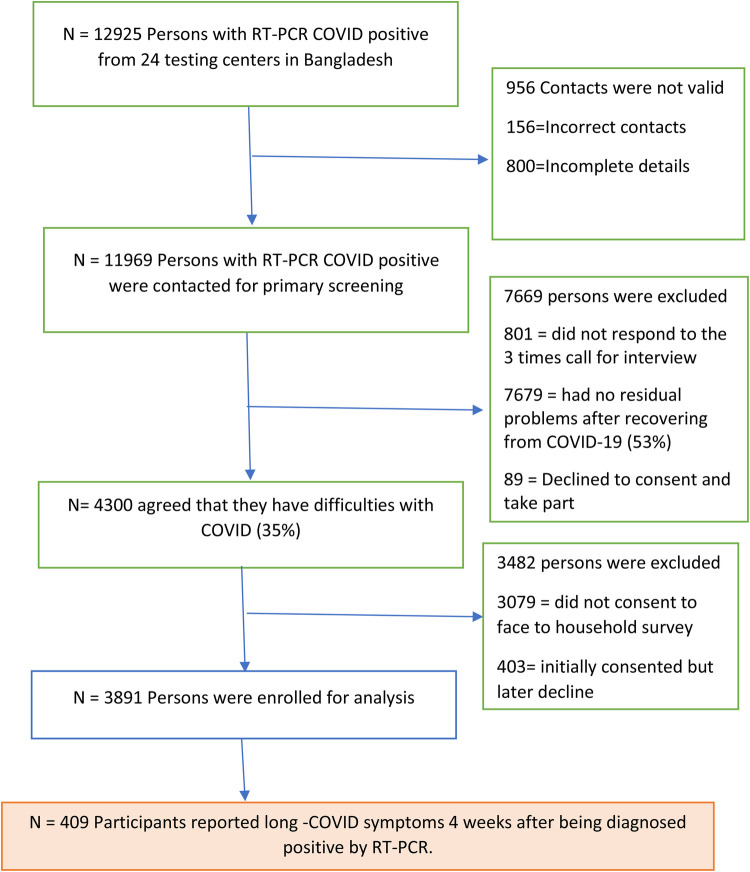
STROBE flow diagram of the study.

Figure 1 shows that fatigue was the most frequently reported symptom among participants followed by cough. A notable number of participants also reported m mental health issues such as depression, anxiety, and lapses in concentration.

## Findings from the Yorkshire Rehabilitation Scale

**Figure 2:**
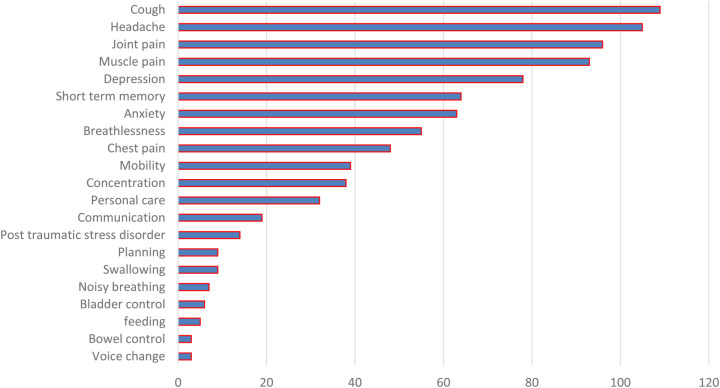
Presentation of post-COVID symptom severity.

The mean post-COVID symptom severity score was 39.46 (SD 16.67) out of 100, where the lowest score was 10 and the highest score was 79. According to the C19-YRS, the mean functional disability score was 18.43 (SD 9.23) out of 60, where the score range was 0–46. Overall health is assessed on a 10-point scale, with 10 representing good health and 0 representing poor health. In the current study, the mean score was 5.17, with scores ranging from 0 to 10. The skewness values of all three scores were calculated to see the consistency of the data, i.e., 0.385, 0.339, and 0.208, respectively. All data are described in Table 2 and illustrated in Figure 2.

**Table 2:** Tabulation of the C19-YRS score.

Subscales (scale range)	Mean	SD	Score range	Skewness
Symptom severity (0–100)	39.46	16.67	10–79	0.385
Functional disability (0–50)	18.43	9.23	0–46	0.339
Overall health 0–10 (0–10)	5.17	2.08	0–10	0.208

## C19-YRS subscale scores

The post-COVID symptom severity mean score was 39.46 (SD 16.67), and the mean functional disability score was 18.43. The mean overall health score was 5.17 on the C19-YRS subscale. The skewness values were 0.385, 0.339, and 0.208.

## Correlation between C19-YRS subscales

An association test using Pearson’s correlation was conducted between the C19-YRS subscales and overall health score. The results revealed a positive association between symptom severity and functional disability, a moderately negative association between symptom severity and overall health, and a negative association between overall health and functional disability. The Pearson correlation results are demonstrated in Table 3.

**Table 3:** Correlation of the C19-YRS subscale with the overall health scale.

Pearson’s correlation (significance) across subscale			
	Symptom severity	Functional disability	Overall health
Symptom severity	1		
Functional disability	0.889** (<0.001)	1	
Overall health	−0.658** (<0.001)	−0.648** (<0.001)	1

** Correlation significant at the 0.01 level (two-tailed).

## Discussion

To determine the level of rehabilitation that was necessary for persons who have been experiencing long-term symptoms of COVID-19, the purpose of this study was to utilize the COVID-19 Yorkshire Rehabilitation Scale (C19-YRS). Based on the findings, the C19-YRS screening test can identify the key chronic medical issues that patients who have recovered from COVID-19 may now be experiencing (11). By employing this metric, we were able to establish a connection between the severity of various symptoms and the underlying serious disease. Through the utilization of this scale, we were able to ascertain that the severity of a wide variety of distinct symptoms was associated with the severity of the underlying disease. It was determined that the severity of the symptoms could be classified into three separate phenotypes: mild, moderate, and severe. According to the Pearson correlation test results, the severity of the symptoms that were encountered within each group was comparable to one another. The results of the test showed that there was a significant positive connection (0.889) between the severity of symptoms and functional difficulty and that there was a somewhat negative correlation (−0.648) between the severity of symptoms and the overall health status. When the severity categories were taken into consideration, the average score likewise suggested that there was a similar association between the severity of symptoms and the level of functional impairment. When it came to functional disability, the average score for the light group was 11, while the score for the intermediate category was 19, and the score for the intense category was 35. One possible indication that the symptoms of post-COVID syndrome are caused by a common underlying pathophysiological mechanism was the fact that the severity of specific symptoms is consistent across all of the groups. PCS was characterized by a number of significant processes, including vascular damage (hypercoagulability) (12, 13), immunological dysregulation (13), and dysautonomia (14, 15). Preexisting research offers evidence of the prevalence of these processes (16). Future research should concentrate on identifying the underlying factors that are responsible for the great variation in symptom presentations that can be observed among individuals. The World Health Organization (WHO) has developed a classification system known as the International Classification of Functioning, Disability, and Health (ICF) that provides a systematic framework for understanding the relationships that exist between the many aspects of a health condition (WHO 2001).

## Conclusion

The C19-YRS covers the multiple body systems affected by COVID-19 and covers all domains of the ICF (WHO) framework. The findings of this study indicate a strong link between long-term symptoms and functional disability. By using the C19-YRS screening tools, symptom severity can be classified. The classification of symptom severity is an ideal resource for policymakers and health systems to decide upon rehabilitation intervention. Further research is needed to understand the common mechanisms and pathophysiological basis of post-COVID symptoms.

## Data Availability

The original contributions presented in the study are included in the article/Supplementary Material, further inquiries can be directed to the corresponding author.
